# Analysis of Medicare Advantage Plans’ Supplemental Benefits and Variation by County

**DOI:** 10.1001/jamanetworkopen.2021.14359

**Published:** 2021-06-23

**Authors:** Hannah L. Crook, Aaron T. Zhao, Robert S. Saunders

**Affiliations:** 1 Duke-Margolis Center for Health Policy, Duke University, Durham, North Carolina; 2 Trinity College of Arts and Sciences, Duke University, Durham, North Carolina; 3 Duke-Margolis Center for Health Policy, Duke University, Washington, DC

## Abstract

This cross-sectional study examines the uptake of several newly allowable Medicare Advantage benefits in 2021 as well as geographic differences in benefit offerings between rural and urban areas, plan penetration, and social vulnerability.

## Introduction

Because of the 2018 Creating High-Quality Results and Outcomes Necessary to Improve Chronic (CHRONIC) Care Act^1^ and complementary guidance by the Centers for Medicare & Medicaid Services (CMS),^2^ Medicare Advantage (MA) plans have the flexibility to offer new supplemental benefits to address enrollees’ broader health and social needs, but few plans have taken advantage of these flexibilities to date.^3,4^ The categories of new benefits that can be offered include primarily health-related benefits, such as adult day health services, home-based palliative care, in-home support services, caregiver support, and therapeutic massage, as newly allowable examples in CMS guidance; special supplemental benefits for the chronically ill (SSBCI); and special COVID-19 supplemental benefits. We examined the uptake of several newly allowable benefits in 2021 as well as geographic differences in benefit offerings between areas by urbanicity, MA penetration, and social vulnerability. We also sought to determine whether counties with higher levels of urbanicity, MA penetration, and social vulnerability were more likely to have a plan offering a newly allowable supplemental benefit.

## Methods

In this cross-sectional study, beginning in October 2020, we combined CMS publicly available contract year 2019 to 2021 plan benefit package data^5^ on new primarily health-related supplemental benefits, SSBCI, and COVID-19 supplemental benefits with 2021 CMS data on MA penetration, 2010 census data on county urbanicity, and 2018 US Centers for Disease Control and Prevention social vulnerability index data. For each analysis, we excluded dual-eligible special needs plans, Medicare-Medicaid plans, and Programs of All-Inclusive Care for the Elderly plans, as they are governed by different regulations. We excluded Puerto Rico from the geographic analyses. Per the Duke University institutional review board, this study did not require review nor informed consent because it used publicly available nonhuman participant data. This report followed the guidelines outlined by Strengthening the Reporting of Observational Studies in Epidemiology (STROBE) for cross-sectional studies.^6^ We compared the characteristics of counties (eg, urbanicity, MA penetration) where plans offered a newly allowable benefit vs counties without such a plan using χ^2^ tests with 2-tailed statistical significance of *P* < .05 using JMP Pro version 15 (SAS Institute).

## Results

As summarized in the Table, 583 of 5754 plans (10.1%) in 2021 offered at least 1 of the new primarily health-related supplemental benefits in 2021; these plans covered 2 231 428 of 22 873 411 beneficiaries (9.8%). Additionally, 1271 plans (22.1%) offered a supplemental benefit specific to COVID-19, such as care packages or face masks, and 637 plans (11.1%) offered SSBCI.

**Table.** Plans Offering Selected Categories of New Supplemental Benefits Over Time^a^

Supplemental benefit	Plans/beneficiaries (% of plans /% of beneficiaries)^b^		
	2019: Early implementation, 4489 plans; 20 190 283 beneficiaries	2020: Full chronic care act implementation, 5215 plans; 21 908 934 beneficiaries	2021: 1 y After full implementation, 5754 plans; 22 873 411 beneficiaries
New primarily health-related supplemental benefits			
Adult day health services	0/0	63/444 548 (1.2/2)	88/478 461 (1.5/2.1)
Home-based palliative care	23/178 347 (0.5/0.9)	58/388 636 (1.1/1.8)	128/561 873 (2.2/2.5)
In-home support services	71/266 494 (1.6/1.3)	148/802 958 (2.8/3.7)	298/1 263 500 (5.2/5.5)
Caregiver support	389/3 460 204 (8.7/17.1)	77/413 479 (1.5/1.9)	87/502 588 (1.5/2.2)
Therapeutic massage (nonopioid pain management before 2020)	24/18 605 (0.5/0.1)	189/699 244 (3.6/3.2)	158/337 697 (2.7/1.5)
Any new primarily health-related supplemental benefit above	507/3 923 650 (11.3/19.4)	360/1 617 519 (6.9/7.4)	583/2 231 428 (10.1/9.8)
COVID-19 benefits^c^	NA	NA	1271/5 590 495 (22.1/24.4)
SSBCI	NA	201/956 326 (3.9/4.4)	637/2 161 692 (11.1/9.5)

Abbreviations: NA, not applicable; SSBCI, Special Supplemental Benefits for the Chronically Ill.

^a^ Table excludes dual-needs special needs plans, Medicare-Medicaid plans, and Programs of All-Inclusive Care for the Elderly plans.

^b^ Beneficiary numbers are of December 2019, December 2020, and January 2021.

^c^ Only available to plans for 2021.

We found differences in mean urbanicity, MA penetration, and social vulnerability index between counties with and without a new primarily health-related supplemental benefit offering. Counties with an MA plan offering 1 of the selected benefits, compared with counties not offering any selected benefits, were more urban (mean [interquartile range {IQR}] urbanicity, 48% [24%-74%] vs 35% [0%-60%]; *P* < .001) and had higher mean (IQR) MA penetration (40% [33%-48%] vs 25% [11%-36%]; *P* < .001). Selected benefits were offered in areas with slightly higher mean (IQR) scores on the social vulnerability index (0.52 [0.30-0.73] vs 0.48 [0.20-0.77]; *P* = .002).

The Figure highlights the geographic spread of selected primarily health-related supplemental benefits, showing the limited geographic reach of the these benefits and the larger coverage for COVID-19 supplemental benefits. A small number of plans offered SSBCI benefits nationwide but were only available to enrollees with certain chronic conditions.

**Figure.**
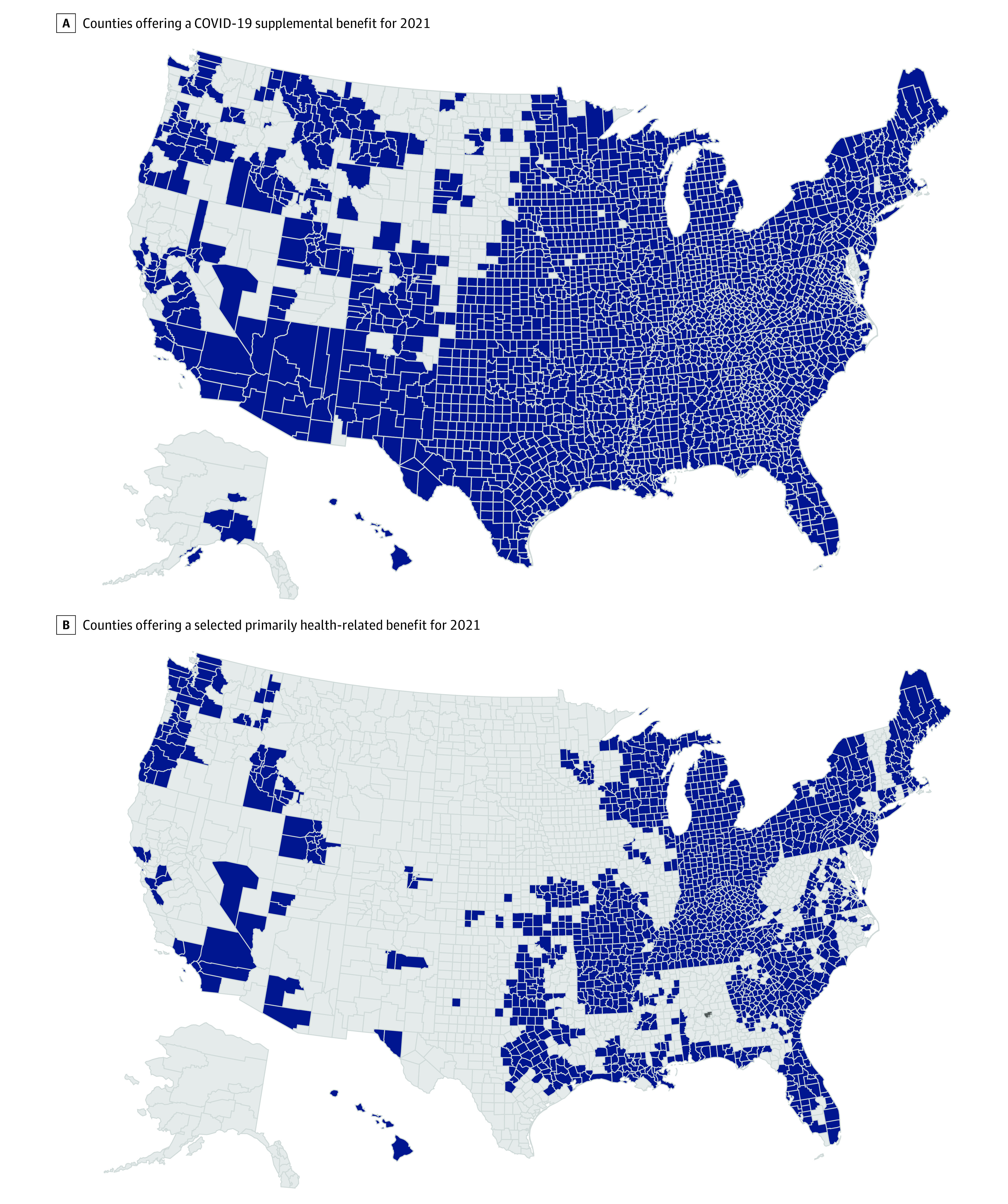
Counties Offering COVID-19 Supplemental Benefits or a Primarily-Health Related Benefit in 2021 Blue shading indicates counties offering a benefit.

## Discussion

New supplemental benefits can be a tool to address MA beneficiaries’ health and social needs. However, only a relatively small proportion of MA plans (10.1%) are taking advantage of the most complex benefits. Uptake of COVID-19 benefits illustrates how supplemental benefits can be a tool for the Biden administration’s priorities on equity, social drivers of health, and COVID-19, but prior qualitative research has identified multiple challenges that will slow new benefits offerings, such as few community partners in rural areas, logistical challenges in setting up any new benefit, strategic trade-offs given no new funding, and limited evidence on implementation and how to target new benefits.^3^ Several strategies could improve future supplemental benefit uptake and spread to new areas, such as additional regulatory clarity on what constitutes a primarily health-related benefit, alignment with other value-based payment or value-based insurance design efforts, and more implementation research on social drivers benefits. Study limitations include the lack of standardized naming conventions for benefits prior to 2020, which may lead to an undercounting of benefits in 2019. Additionally, plan benefit offerings may not be reflective of beneficiary use, as beneficiaries may not qualify for or be aware of the benefit.
